# Refractive assessment by wavefront aberrometry compared to subjective refraction in PRK patients

**DOI:** 10.1007/s00417-025-06868-9

**Published:** 2025-06-25

**Authors:** Amr Saad, Andreas Frings

**Affiliations:** 1 Department of Ophthalmology, Heinrich Heine University, Moorenstraße 5, Duesseldorf, 40225 Germany; 2 Department of Ophthalmology, Stadtspital Zürich, Zurich, Switzerland; 3 Spross Research Institute, Zurich, Switzerland

**Keywords:** Refractive surgery, Photorefractive keratectomy, Phototherapeutic keratectomy, Wavefront aberrometry, Subjective refraction

## Abstract

**Purpose:**

To compare wavefront aberrometry (WA) refraction with subjective refraction (SR) before and after transepithelial photorefractive keratectomy (tPRK) or combined phototherapeutic keratectomy (PTK)-PRK.

**Methods:**

In our monocentric retrospective study, we aimed to compare refraction measurements obtained using wavefront aberrometry (WA) with subjective refraction (SR) for 154 eyes that underwent PRK treatment. The eyes underwent either tPRK treatment with the Amaris750 excimer laser or combined PTK-PRK treatment with the MEL90 excimer laser. Preoperative spherical equivalent, age, and sex were matched between the two groups. Wavefront measurements were performed with Sirius in tPRK patients and with WASCA in PTK-PRK patients. Follow-up was 6 months postoperatively. We used Bland-Altman plots and intraclass coefficient (ICC) analysis to demonstrate the agreement of SR and WA refraction.

**Results:**

Preoperatively, there was a high agreement between WA and SR refraction in both treatment groups. However, postoperatively, there was almost no agreement between the two methods (ICC = 0). WA refraction provided more significant hyperopic refraction values postoperatively, while the agreement for cylinder values was lower compared to the sphere.

**Conclusion:**

Our study shows that the choice of refraction measurement method should be carefully considered in PRK patients, particularly in the postoperative period and when using aberrometry systems. Further research with larger sample sizes is needed to fully investigate this topic.

## Abstract (highlights)

**Key messages:**

*****What is known***:**

- Wavefront aberrometry (WA) and subjective refraction (SR) are two common methods used to measure refractive error in patients undergoing refractive surgery.

*****What is new***:**

- Postoperatively, there was almost no agreement between WA and SR (ICC = 0) in tPRK and PTK-PRK patients.
- WA refraction showed more hyperopic values and less agreement for cylinder compared to sphere.

## Introduction

Accurate measurement of refractive errors is crucial in the preoperative evaluation to achieve optimal visual outcomes in refractive surgery. Different methods can be used for refractive assessment, including subjective (manifest subjective refraction) and objective (autorefraction, wavefront aberrometry) techniques [1]. While subjective refraction (SR) is considered the gold standard [2], it is a time-consuming and subjective process. Wavefront aberrometers (WA) can provide information not only on low-order aberrations, such as sphere and cylinder, but also on higher-order aberrations, such as coma and trefoil [3, 4], potentially leading to more precise and customized treatment approaches. While there is an array of wavefront aberrometers available for clinical use [5, 6], there remains a disagreement between objective and subjective values [7] as well as a tendency for wavefront aberrometers to produce more myopic values than autorefractometers [8]. Various studies have compared wavefront aberrometry and autorefraction in refractive surgery patients, but the results have been inconsistent and only few have focused specifically on PRK cohorts, highlighting the novelty of our approach [1, 9]. Discrepancies between WA and SR measurements can have significant clinical implications, such as affecting treatment planning and evaluating surgical outcomes. Ultimately, these potential shifts could impact patient satisfaction and visual quality after refractive surgery.

The aim of our retrospective analysis is to compare the pre- and postoperative measurements of two methods, WA and SR, in patients who underwent photorefractive keratectomy (PRK). PRK can be performed in either a one-step or two-stage procedure, with the former involves ablating the epithelium and stroma simultaneously. Our study focuses on comparing the pre- and postoperative measurements of patients who underwent one-step transepithelial PRK (tPRK) and two-step phototherapeutic keratectomy (PTK)-PRK. We aim to identify any differences between the two methods and provide guidance to surgeons on the interpretation of refraction values, especially in the context of the increasing use of WA measurements. To our knowledge, no previous studies have compared the agreement between WA refraction obtained from Sirius and WASCA with conventional SR. Given that our study focuses on a PRK cohort, our novel approach has significant implications for refractive surgeons. Thus, additional research is necessary to improve refractive assessment before and after refractive surgery.

## Materials and methods

In this study, we retrospectively analyzed refraction data from 154 eyes of 86 patients who underwent treatment in private practices in Germany. The study received approval from the local research ethics committee of the University of Duesseldorf and was conducted in accordance with the Declaration of Helsinki. It was also registered on drks.de database (DRKS-ID: DRKS00030980). Prior to participation, all patients provided written informed consent for the use of their routinely collected data for research purposes. The data used for this research project were collected from January to February 2023 and the author did not have access to any information that could identify individual participants during or after data collection.

Two treatment groups were included in this retrospective study: Group A received conventional tPRK treatment with the Amaris750 excimer laser (Schwind eye-tech solutions, Kleinostheim, Germany), a high-performance laser with a 750 Hz repetition rate, while Group B underwent the combined PTK-PRK method using the MEL90 laser (Carl Zeiss Meditec, Oberkochen, Germany) as described in previous publication [10]. The latter eyes underwent sequential PTK treatment for epithelial ablation followed by refractive ablation of the stroma in the PRK mode. To ensure an unbiased sample, we randomly selected 154 eyes of 86 patients from a larger cohort of approximately 197 eyes, using the random number function in MS Excel (Microsoft Excel 2017, Microsoft^®^). Inclusion criteria for PRK surgery were age above 18 years, absence of ocular disease, prior ocular surgery or trauma, or systemic disorders affecting the eye. Only eyes with a preoperative spherical equivalent (SE) of −1 diopter (D) or greater were included, to focus on myopic patients from the real-world population and ensure clinical relevance to typical PRK candidates. Patients with any systemic diseases that could affect the eye were excluded. The surgeries were performed by the same surgeon (A.F.) under topical anesthesia. The optical zone and transition zone were set to 6.5 mm and 1.5 mm, respectively, for all eyes. The PRK treatment followed our own ablation nomogram without using a wavefront-guided mode. At the end of the procedure, Mitomycin C (MMC, 0.02%) was applied for 15–30 s depending on ablation depth.

Preoperative assessments were performed according to a standard internal protocol. These assessments included non-cycloplegic manifest subjective refraction (SR) and wavefront aberrometry (WA). The SR was conducted first, followed by the WA scans, which were performed by the same certified optometrist to minimize inter-examiner bias. To evaluate subjective refraction, we obtained uncorrected distance visual acuity (UDVA) and corrected distance visual acuity (CDVA), 2 weeks before and 6 months after surgery. In addition, we collected topography data using Schwind Anterior Segment Analyzers (Peramis and Sirius) for tPRK patients and WASCA aberrometer (Carl Zeiss, Oberkochen, Germany) for PTK-PRK patients. To ensure high repeatability, we obtained three consecutive measurements for WA assessments and calculated the mean, reducing any operator-related bias. All measurements were obtained under standardized light conditions and patient’s instructions [11].

The WASCA device from Carl Zeiss Meditec, is based on a Hartmann-Shack sensor and utilizes a laser light beam reflected at the fovea to create a wavefront that is detected by a charged-couple device (CCD) camera [12, 13]. The Peramis (Schwind eye-tech-solutions, Kleinostheim, Germany) combines topography and aberrometry in one device and analyzes the corneal wavefront using a pyramid wavefront sensor.

The study analyzed the agreement between subjective refraction (SR) and wavefront aberrometry (WA) pre- and postoperatively in both treatment groups. The agreement between WA and manifest refraction was assessed using a standard Bland-Altman plot and the Intraclass Correlation Coefficient (ICC) was estimated using a two-way mixed effects model for absolute agreement and single rater/measurement. The corresponding definition of the selected ICC was described by Koo et al. [14]. All statistical analysis was performed using the R Core Team software (R Foundation for Statistical Computing 2021, Vienna, Austria).

## Results

In this study, we analyzed 154 eyes from 86 patients who were matched for preoperative refraction, age, and gender. Postoperatively, no complications were reported. Among the subjects, 36.6% were male and 63.4% were female, with a mean age of 35 years (range: 21–53 years).

The refractive values obtained from the two assessment methods are summarized in Tables 1 and 2. Preoperatively, in the overall cohort, the spherical equivalent (SE) ranged from − 1.00 diopter (D) to −7.38 D for SR and from − 0.95 D to −7.36 D for WA. After surgery, the ranges were − 0.5 D to 0.25 D and − 0.95 D to 1.41 D for SR and WA, respectively.

**Table 1:** Preoperative descriptive data: wavefront aberrometry and subjective refraction

Parameter	tPRK (*N* = 77)	PTK-PRK (*N* = 77)	Total (*N* = 154)
WA Sphere (D)			
Range	−1.17, −7.50	−1.26, −7.39	−1.17, −7.50
Mean (SD)	−4.28 (1.84)	−4.23 (1.84)	−4.26 (1.83)
Median (Q1, Q3)	−4.01 (−2.74, −6.06)	−3.89 (−2.72, −6.05)	−3.95 (−2.73, −6.06)
WA Cylinder (D)			
Range	−1.69, −0.02	−1.77, −0.02	−1.77, −0.02
Mean (SD)	−0.47 (0.38)	−0.46 (0.39)	−0.47 (0.39)
Median (Q1, Q3)	−0.32 (−0.56, −0.21)	−0.33 (−0.51, −0.21)	−0.33 (−0.55, −0.21)
WA Spherical Equivalent (D)			
Range	−0.95, −7.36	−1.04, −7.17	−0.95, −7.36
Mean (SD)	−4.04 (1.84)	−4.00 (1.85)	−4.02 (1.84)
Median (Q1, Q3)	−3.64 (−2.46, −5.81)	−3.63 (−2.40, −5.73)	−3.63 (−2.42, −5.80)
Manifest Sphere (D)			
Range	−1.25, −7.50	−1.50, −7.50	−1.25, −7.50
Mean (SD)	−4.31 (1.77)	−4.29 (1.75)	−4.30 (1.76)
Median (Q1, Q3)	−4.00 (−2.75, −6.25)	−4.25 (−3.00, −6.00)	−4.00 (−3.00, −6.00)
Manifest Cylinder (D)			
Range	−1.75, 0.00	−1.75, 0.00	−1.75, 0.00
Mean (SD)	−0.45 (0.41)	−0.45 (0.45)	−0.45 (0.43)
Median (Q1, Q3)	−0.25 (−0.75, −0.20)	−0.25 (−0.75, 0.00)	−0.25 (−0.75, 0.00)
Manifest Spherical Equivalent (D)			
Range	−1.00, −7.38	−1.25, −7.00	−1.00, −7.38
Mean (SD)	−4.09 (1.81)	−4.06 (1.78)	−4.08 (1.79)
Median (Q1, Q3)	−3.88 (−2.62, −5.88)	−4.00 (−2.75, −5.88)	−3.88 (−2.62, −5.88)

**Table 2:** Postoperative descriptive data: wavefront aberrometry and subjective refraction

Parameter	tPRK (*N* = 77)	PTK-PRK (*N* = 77)	Total (*N* = 154)
WA Sphere (D)			
Range	−0.61, 1.50	−0.54, 1.48	−0.61, 1.50
Mean (SD)	0.37 (0.37)	0.35 (0.39)	0.36 (0.38)
Median (Q1, Q3)	0.35 (0.12, 0.61)	0.32 (0.10, 0.64)	0.33 (0.11, 0.63)
WA Cylinder (D)			
Range	−0.67, 0.00	−0.95, 0.00	−0.95, 0.00
Mean (SD)	−0.19 (0.12)	−0.19 (0.14)	−0.19 (0.13)
Median (Q1, Q3)	−0.18 (−0.26, −0.10)	−0.19 (−0.26, −0.11)	−0.19 (−0.26, −0.11)
WA Spherical Equivalent (D)			
Range	−0.95, 1.41	−1.02, 1.38	−1.02, 1.41
Mean (SD)	0.27 (0.37)	0.25 (0.40)	0.26 (0.38)
Median (Q1, Q3)	0.27 (0.01, 0.50)	0.20 (−0.01, 0.51)	0.24 (0.00, 0.50)
Manifest Sphere (D)			
Range	−0.50, 0.25	−0.50, 0.25	−0.50, 0.25
Mean (SD)	−0.08 (0.18)	−0.19 (0.20)	−0.14 (0.19)
Median (Q1, Q3)	0.00 (−0.25, 0.00)	−0.25 (−0.25, 0.00)	0.00 (−0.25, 0.00)
Manifest Cylinder (D)			
Range	−0.25, 0.00	−0.50, 0.00	−0.50, 0.00
Mean (SD)	−0.11 (0.13)	−0.14 (0.16)	−0.13 (0.14)
Median (Q1, Q3)	0.00 (−0.25, 0.00)	0.00 (−0.25, 0.00)	0.00 (−0.25, 0.00)
Manifest Spherical Equivalent (D)			
Range	−0.50, 0.25	−0.62, 0.25	−0.62, 0.25
Mean (SD)	−0.13 (0.16)	−0.26 (0.19)	−0.20 (0.19)
Median (Q1, Q3)	−0.12 (−0.25, −0.06)	−0.25 (−0.38, −0.12)	−0.25 (−0.25, −0.12)

**Fig. 1:**
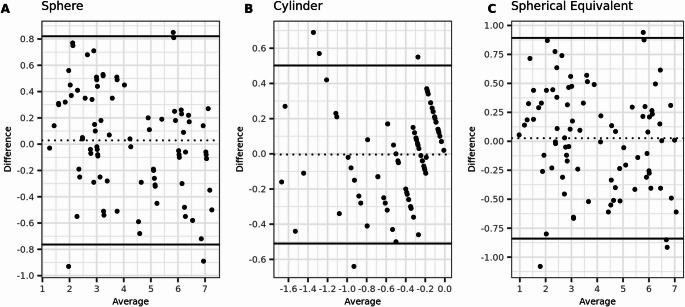
Bland-Altmann plots for preoperative agreement of sphere, cylinder, and spherical equivalent (SE) measured with subjective refraction (SR) and wavefront aberrometry (WA). The dashed lines indicate the mean difference, and the limits of agreement (LoA) are depicted by the two solid lines. (*N* = 154 eyes)

**Fig. 2:**
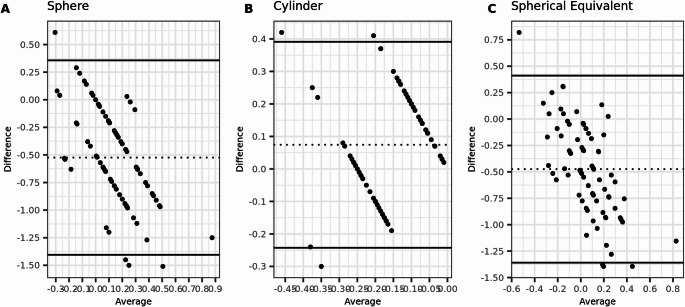
Bland-Altmann plots for postoperative agreement of sphere, cylinder, and spherical equivalent (SE) measured with subjective refraction (SR) and wavefront aberrometry (WA). The dashed lines indicate the mean difference, and the limits of agreement (LoA) are depicted by the two solid lines. (*N* = 154 eyes)

The preoperative agreement between subjective refraction and wavefront aberrometry was high, with an ICC and correlation coefficient (r_s_) of 0.97 each for SE. The mean difference was small (0.03 ± 0.44 D), but the Limits of Agreement were large (more than 1 D). However, postoperatively, there was almost no agreement between the two methods, with an ICC of zero. The results are presented in Tables 3 and 4. Additionally, the Bland-Altman plots illustrate the agreement between the two methods, both preoperatively and postoperatively (Figs. 1 and 2).

**Table 3:** Agreement of preoperative refraction measurements with WA and SR

	Sphere	Cylinder	Spherical Equivalent
Mean Difference ± SD	0.03 ± 0.4	0 ± 0.26	0.03 ± 0.44
(95%CI)	(−0.06 to 0.12)	(−0.06 to 0.05)	(−0.07 to 0.12)
Range	−0.93 to 1.00	−0.64 to 1.00	−1.08 to 1.00
95% limits of agreement	−0.76 to 0.82	−0.51 to 0.50	−0.84 to 0.89
(1.96 × SD of difference)	(0.78)	(0.5)	(0.85)
ICC	0.97	0.82	0.97
(95%CI)	(0.96 to 0.98)	(0.73 to 0.88)	(0.95 to 0.98)
Correlation rs	0.97	0.82	0.97
(95%CI)	(0.96 to 0.98)	(0.73 to 0.88)	(0.95 to 0.98)

**Table 4:** Agreement of postoperative refraction measurements with WA and SR

	Sphere	Cylinder	Spherical Equivalent
Mean Difference ± SD	−0.52 ± 0.45	0.07 ± 0.16	−0.47 ± 0.45
(95%CI)	(−0.63 to −0.42)	(0.04 to 0.11)	(−0.58 to −0.37)
Range	−1.51 to 1.00	−0.30 to 0.00	−1.40 to 1.00
95% limits of agreement	−1.40 to 0.36	−0.24 to 0.39	−1.36 to 0.41
(1.96 × SD of difference)	(0.86)	(0.31)	(0.87)
ICC	0	0.16	0
(95%CI)	(0.00 to 0.07)	(0.00 to 0.36)	(0.00 to 0.08)
Correlation rs	0	0.16	0
(95%CI)	(0.00 to 0.07)	(0.00 to 0.36)	(0.00 to 0.08)

**Fig. 3:**
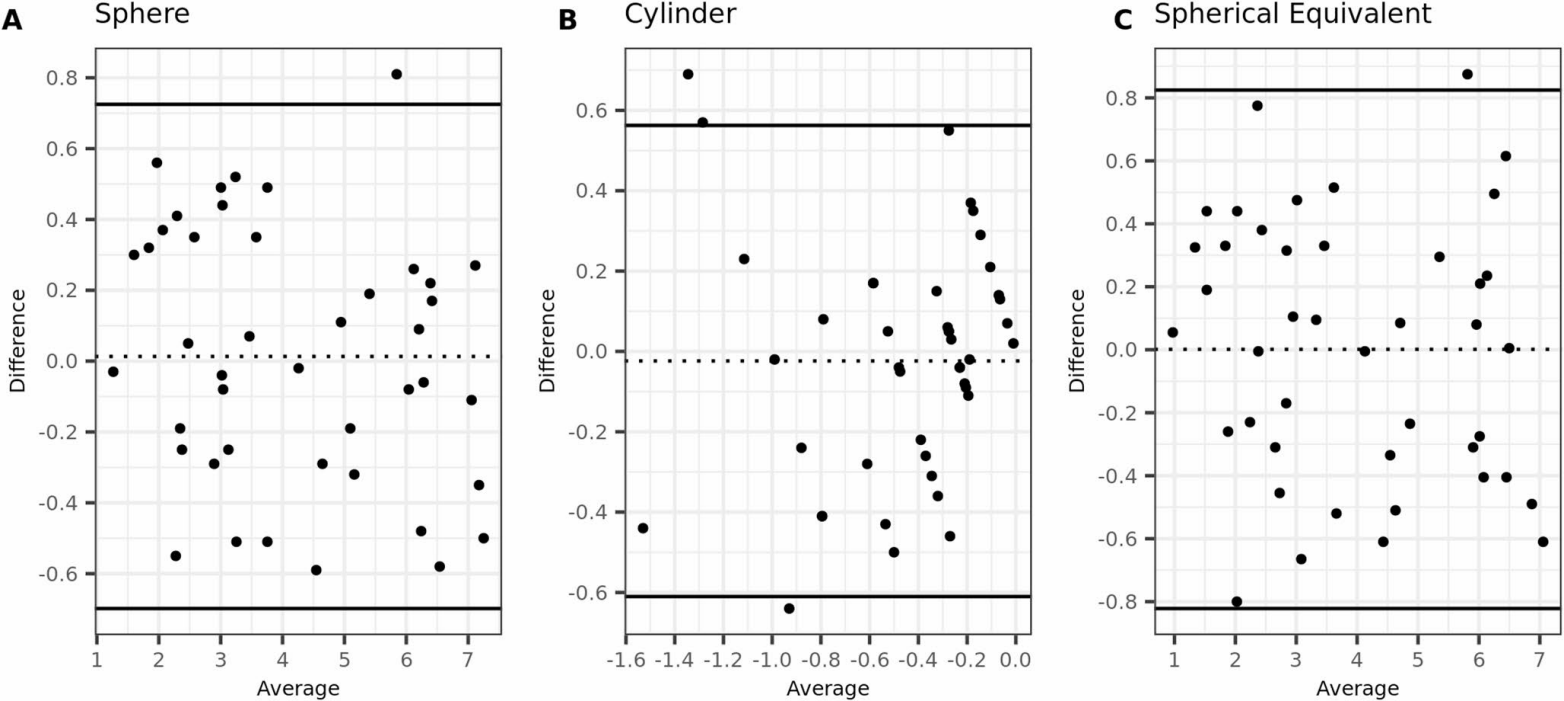
Bland-Altmann plots for preoperative agreement of sphere, cylinder, and spherical equivalent (SE) measured with subjective refraction (SR) and wavefront aberrometry (WA) in the tPRK group. The dashed lines indicate the mean difference, and the limits of agreement (LoA) are depicted by the two solid lines. (*N* = 77 eyes)

**Fig. 4:**
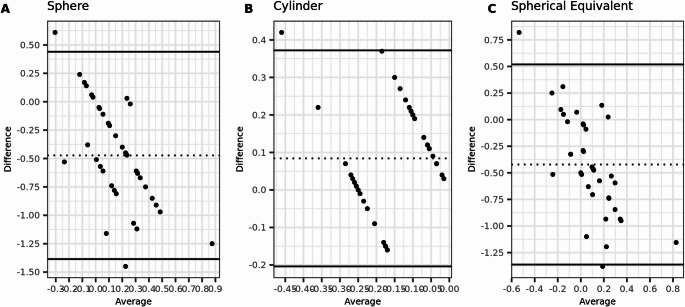
Bland-Altmann plots for postoperative agreement of sphere, cylinder, and spherical equivalent (SE) measured with subjective refraction (SR) and wavefront aberrometry (WA) in the tPRK group. The dashed lines indicate the mean difference, and the limits of agreement (LoA) are depicted by the two solid lines. (*N* = 77 eyes)

**Fig. 5:**
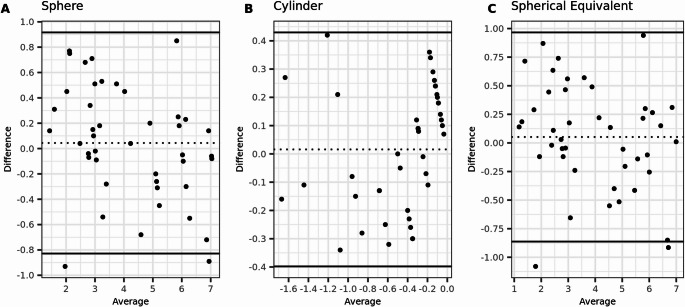
Bland-Altmann plots for preoperative agreement of sphere, cylinder, and spherical equivalent (SE) measured with subjective refraction (SR) and wavefront aberrometry (WA) in the PTK-PRK group. The dashed lines indicate the mean difference, and the limits of agreement (LoA) are depicted by the two solid lines. (*N* = 77 eyes)

**Fig. 6:**
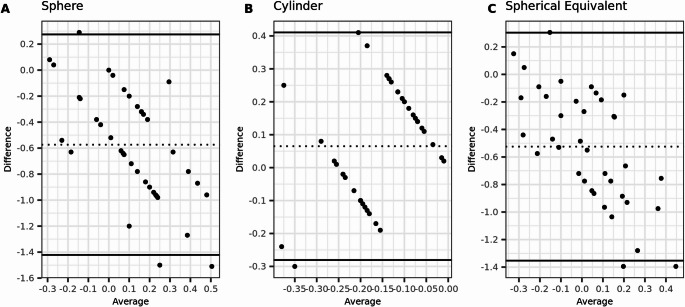
Bland-Altmann plots for postoperative agreement of sphere, cylinder, and spherical equivalent (SE) measured with subjective refraction (SR) and wavefront aberrometry (WA) in the PTK-PRK group. The dashed lines indicate the mean difference, and the limits of agreement (LoA) are depicted by the two solid lines. (*N* = 77 eyes)

Furthermore, there were no significant differences in agreement between the tPRK and PTK-PRK treatment groups, both preoperatively and postoperatively, as indicated by the alignment of the 95% confidence intervals (95% CI) for all agreement parameters (Figs. 3, 4, 5 and 6).

## Discussion

Refractive surgery is becoming increasingly popular, with PRK being one of the preferred corneal refractive treatments [15]. Accurate refractive assessment of refractive candidates is crucial to achieve optimal outcomes. The comparison of WA refraction and SR in PRK patients has been limited to only a few studies [1, 16]. With the increasing use of wavefront aberrometers and wavefront-guided ablation, it is essential to also analyze and compare this method with the traditional refraction assessments. In this study, we aim to contribute to this important topic by examining wavefront aberrometry refraction using WASCA and Sirius in PRK patients.

Wavefront aberrometers (WA) have become an important tool in the evaluation of refractive patients due to their ability to detect wavefront errors that affect visual acuity [17]. However, there are differences in the refraction values obtained with WA compared to other methods such as autorefraction (AR) and subjective refraction (SR). Some WA, such as WASCA, tend to produce more myopic values due to instrumental myopia [8], while others, such as Zeiss i.ProfilerPlus (Carl Zeiss, Oberkochen, Germany), achieve comparable results to AR and SR [9]. Despite these differences, some studies have reported good agreement between WA refraction and SR [18]. The main reason for these differences is that WA use different optical metrics to calculate refractive error [19], but they are not always clinically relevant [20]. The fact that there is a technological gap between WASCA, an older Hartmann-Shack-based device that is no longer in production, and the newer Peramis system may have contributed to the observed postoperative discrepancies between WA and SR.

Various factors may cause differences in the agreement of the different methods. For example, measurement errors, calibration issues or variations in the patient’s fixation may contribute to alignment differences [21]. In addition, differences in the optical properties such as tear film abnormalities, corneal or lenticular opacities, small pupil size, and abnormalities of the vitreous and retina must be considered when evaluating measurements and their differences. Standardizing room illumination can reduce pupil diameter fluctuations when obtaining measurements [22]. It is worth highlighting that wavefront measurements can distinctly differentiate between the spherical and cylindrical components of refractive error and higher-order aberrations (HOAs), which affect manifest refraction as well. This capability of wavefront measurements to clearly distinguish between these components can help improve the accuracy and reliability of refractive measurements and enable more precise diagnosis and treatment of refractive errors.

The current data highlights a difference in alignment between sphere and cylinder measurements, indicating a slightly better agreement in sphere measurements as compared to cylindrical values. This is consistent with the observations of a prior study by Bamdad et al. [21], which reported good agreement in sphere measurements and poor correlation in cylinder measurements. The lower agreement in cylinder measurements may be attributed to the greater complexities of measuring cylinder accurately.

Our study revealed good agreement between preoperative measurements, with the exception of a wide range of variability expressed in the 95% limits of agreement (LoA), which may not meet clinical acceptability standards. In contrast, there was almost no agreement postoperatively. Previous research has indicated that the deviation between autorefraction (AR) and subjective refraction (SR) after photorefractive keratectomy (PRK) surgery is likely related to the wound healing process, as demonstrated by Oyo-Szerenyi et al. [23] and confirmed by Rosa et al. [24], who reported good agreement between AR and SR preoperatively, but only poor agreement after PRK surgery. The degree of postoperative difference between SR and objective measurements, including AR and wavefront aberrometry (WA), is believed to depend on the amount of refractive correction. Thus, measurement alignment between SR and WA was shown to be high in eyes with low refractive error and low in eyes with higher refractive errors [25].

Sirius biometric measurements have been found to be in good agreement with other aberrometers such as the VX120 (Visionix Luneau, France) [26]. However, a study by Lanza et al. suggested that Sirius may not provide accurate refraction values after myopic PRK treatment due to the Placido disc measurement bias of post-PRK corneas [27]. It is challenging to compare the results of different wavefront aberrometers with SR or even AR due to the variation in measurement methods and the hardware and software used.

The poor agreement between subjective and objective refraction is not limited to adults but also present in children, highlighting the need for cycloplegia to improve the agreement between them [28]. The difference between subjective and objective refraction tends to increase with higher refractive error, consistent with previous research in adults [1]. Overall, objective WA-based refraction methods have been shown to offer superior accuracy, especially in cylinder measurements, due to their ability to measure actual wavefront aberrations of the eye, while avoiding the potential bias and errors associated with subjective patient feedback.

The described disparities between WA and SR can have a substantial impact on clinical outcomes, particularly in treatment planning and patient management. A hyperopic shift in WA may indicate overcorrection, leading surgeons to avoid enhancements when SR indicates emmetropia or slight myopia and the patient is satisfied. This hyperopic bias in WA measurements can lead to misrepresentation of surgical outcomes, highlighting the necessity for clinicians to inform patients preoperatively about potential discrepancies in the future.

## Conclusion

We conclude that the choice of refraction measurement method is a crucial factor to consider in patients undergoing PRK, particularly during the postoperative period and when using aberrometry systems, as they are sensitive to corneal changes and can lead to inaccurate measurements. In the future, machine learning (ML) could greatly change refractive assessment by providing results that closely resemble SR values, which will help refining postoperative assessments for refractive surgery patients [29]. Research studies demonstrate that ML algorithms, such as random forest and gradient boosting, can accurately predict SR from WA data, thereby reducing errors in refraction measurements and enhancing the reliability postoperative care [30, 31]. The retrospective design and relatively small sample size of 154 eyes are limitations of our study. Therefore, our results should be confirmed by further studies with a larger population of refractive patients and a longer follow-up period.
